# Effectiveness of Echium amoenum on premenstrual syndrome: a randomized, double-blind, controlled trial

**DOI:** 10.1186/s12906-020-03084-2

**Published:** 2020-09-29

**Authors:** Maryam Farahmand, Davood Khalili, Fahimeh Ramezani Tehrani, Gholamreza Amin, Reza Negarandeh

**Affiliations:** 1 Nursing & Midwifery Care Research Center, School of Nursing and Midwifery, Tehran University of Medical Sciences, P.O.Box: 1419733171, Mirkhani St., Tohid Sq, Tehran, Iran; 2 Reproductive Endocrinology Research Center, Research Institute for Endocrine Sciences, Shahid Beheshti University of Medical Sciences, Tehran, Iran; 3 Prevention of Metabolic Disorders Research Center, Research Institute for Endocrine Sciences, Shahid Beheshti University of Medical Sciences, Tehran, Iran; 4 Department of Epidemiology and Biostatistics, Research Institute for Endocrine Sciences, Shahid Beheshti University of Medical Sciences, Tehran, Iran; 5 Department of Pharmacognosy, Faculty of Pharmacy, Tehran University of Medical Sciences, Tehran, Iran

**Keywords:** Herbal medicine, Phytosterol, Echium Ameonum, Starch, Premenstrual syndrome, Premenstrual symptoms screening tool

## Abstract

**Background:**

The present study aimed to evaluate the effect of Echium amoenum (EA) on the severity of premenstrual syndrome (PMS) in comparison with placebo.

**Methods:**

The present study was a randomized double-blind controlled clinical trial. A checklist questionnaire was completed by 120, 18 to 35-year-old, college students. And then, 84 eligible women (20 to 35 years old) were enrolled in the trial; they were randomly assigned to two groups of intervention (EA) and control (placebo), with 42 participants in each group. Participants in the intervention group received 450 mg capsules of EA per day (three times a day) from the 21st day of their menstrual cycle until the 3rd day of their next cycle for two consecutive cycles. The severity of PMS was measured and ranked using the premenstrual symptoms screening tool (PSST). The generalized estimating equation was used to compare the total score of the severity of PMS between the two groups.

**Results:**

Sixty-nine women with regular menstrual cycles suffering from PMS completed the study. The mean scores of the symptoms in the EA group were 35.3 and 16.1 (*P* ≤ 0.001) at baseline and after 2 months, respectively, while the mean scores of the symptoms in the placebo group were 31.0 and 28.3 (*P* = 0.09) at baseline and after 2 months, respectively. The evaluation of the first and the second follow-ups in the intervention group showed that, after being adjusted for age and body mass index (*P ≤* 0.001), the mean scores of the premenstrual syndrome, using GEE analysis, have decreased to 6.2 and 11.6, respectively.

**Conclusion:**

Based on the results, in comparison with the placebo group, EA was found to be more effective in improving the symptoms of PMS, and is highly recommended for treatment of this syndrome.

**Trial registration:**

IRCT2015110822779N3; Registration date: 2015–11–27.

## Background

During recent years, health has become the main priority for women. Nowadays, in addition to the emotional role of women at home, women have been accepting more occupational and social roles in society [1]. Premenstrual Syndrome (PMS) is referred to as the periodic recurrence of a set of physical, psychological, and behavioral variations during the second half of the menstrual cycle, which is prevalent among women of reproductive ages and affects their health [2]. This syndrome with a high prevalence (80–90%) [3] has no clear etiology; however, there are some theories such as sensitivity to hormonal changes or disruption of endogenous opioids during the menstrual cycle, stress, and diet which could be related to its etiology [2, 4–6]. Several therapeutic options have been documented for PMS for instance hormonal and psychotropic drugs, non-steroidal anti-inflammatory drugs, diuretics, surgery, lifestyle changes, and herbal or complementary therapies [2, 4, 7].

Echiuma Amoneum (EA) has been traditionally used as a medicine and Romans were among the first individuals who approved its effectiveness around 300 B.C. [8]. In addition, the famous Greek poet, Homer, argued that EA could be benefitial for people’s mood in general [9]. In Iran, people have been growing Echiuma Amoneum in the mountain areas in the north of the country [10] and it was conventionally believed that EA can have sedative effects on patients which is well-documented in old Persian medical textbooks such as the Qanoon by Avicenna [11].

To date, studies conducted on EA have demonstrated the efficacy of the plant as a sedative and diaphoretic, and also a treatment for cough, sore throat and pneumonia [12, 13]. Cyanidin 3-glucoside, the most common anthocyanin found in the petals of *EA*, has had neuroprotective effects and has traditionally been used as an anxiolytic and antidepressant medicine in Asia [14].

Moreover, it has been recommended as mood enhancement [15] and has been promoted for a variety of its effects as a demulcent, anti-inflammatory [16], antioxidant [17, 18] analgesic, anxiolytic, sedative [19–21] and anticonvulsant [22]. Traditionally, EA was being used for the treatment of hyperactive gastrointestinal, respiratory and cardiovascular disorders [23], regulation of metabolism and the hormonal system [24], and menopause symptoms such as hot flash [25]. Additionally, the results of the studies in Iran context have demonstrated the positive effects of the pharmaceutical components of this medicinal plant which include antimicrobial, antiviral, and anti-inflammatory effects as well as its effects on some psychiatric symptoms such as anxiety disorders, obsession, compulsion, and depression without any severe side effects [26–31].

Given the variety of therapeutic effects of the EA and taking into account the fact that anxiety is the most common symptom of PMS [32], for the first time in the present study, the researchers have examined the effectiveness and safety of the aqueous extract of EA on the severity of PMS.

## Methods

The present study was a randomized double-blind controlled clinical trial (CONSORT guidelines) conducted on college students from Tehran University of Medical Sciences and Tehran University after obtaining approval and confirmation from the ethics committee of Tehran University of Medical Sciences.

The 4th edition of the checklist questionnaire of the Diagnostic and Statistical Manual of Mental Disorders (DSM-IV) was used in this study. After receiving written informed consent, the 11-items checklist was distributed among the college students. According to this questionnaire, the criteria for the diagnosis of PMS include observing at least 5 of the 11 symptoms of PMS 7 days before menstruation and one of the first four symptoms of PMS (1-feeling sad, hopeless, or remarkably depressed; 2- significant anxiety, tension, impatience; 3- significant mood swings such as sudden sadness; 4- continuous visible anger and irritability and increased mood changes) [33]. Eligible students were selected and their written informed consent was obtained. And then, the demographic characteristics questionnaire and premenstrual symptoms screening tool (PSST) were completed by the participants before the initiation of the intervention.

The PSST questionnaire consists of 19 items in two sections: The first section contains 14 items of PMS symptoms, and the second section contains five items evaluating the effects of symptoms on women suffering from PMS. All the items were answered using a 4-point Likert scale (none, mild, average, and severe), which are assigned a score from 0 to 3 (0–1–2-3), respectively [34]. The content validity ratio and content validity index of this questionnaire were calculated to be 0.7 and 0.8, respectively. Moreover, the reliability of the scale was confirmed by a Cronbach’s alpha coefficient of 0.9 [35].

Subsequently, eligible participants were randomly assigned to two study groups, including herbal medicine or EA and the placebo. Random blocks were used for randomization. Since the size of the blocks was set at 4, six sequences were created. Random sequences were made by the statistician using random allocator software. The allocator concealed the block size from the executor; thus, the random allocation of the participants was assured. It must be noted that, based on the method of the study, which was a double-blind clinical trial, neither the executor nor the participants were aware of the type of the consumed drugs. In addition, executors did not participate in data analysis.

After implementing the intervention for two consecutive menstrual cycles, the PSST questionnaire was completed again at the end of each menstrual cycle in order to evaluate the severity of PMS symptoms.

Following a pilot study, the number of samples in each group was calculated to be 32. Given a 30% sample loss probability, a total of 84 samples were selected using the following formula:

$$ \upalpha =0.05,\upbeta =0.1,{Z}_{1-\raisebox{1ex}{$\partial $}\!\left/ \!\raisebox{-1ex}{$2$}\right.}=1.96,{Z}_{1-\beta }=1.28,\updelta =6.2,\mathrm{d}=5,\delta = Standard\ deviation,\mathrm{d}=\mathrm{Effect}\ \mathrm{size}. $$

$$ {\displaystyle \begin{array}{c}n=\frac{2{\left({Z}_{1-\raisebox{1ex}{$\partial $}\!\left/ \!\raisebox{-1ex}{$2$}\right.}+{Z}_{1-\beta }\ \right)}^2{\delta}^2}{d^2}\\ {}n=\frac{2{\left(1.96+1.28\ \right)}^2\ 38.44}{25}\simeq 32\end{array}} $$

Inclusion criteria were as follows: having regular menstruation (menstrual cycles of 21 to 35 days), being 18 to 35 years old with diagnosed PMS, no record of smoking cigarettes or consuming alcoholic beverages, no record of consuming drugs including hormonal, herbal, anticonvulsant or antidepressant medications, and no record of allergic reactions to herbal medicines.

**Fig. 1:**
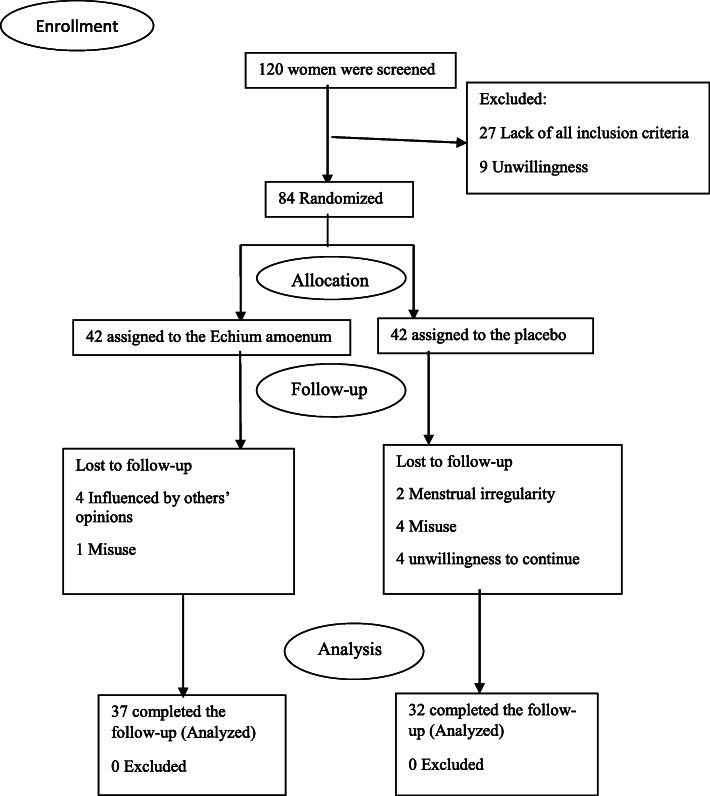
The process of the study

In addition, the following was also considered as the inclusion criteria: no history of psychological diseases or any underlying physical diseases such as diabetes, hypertension, hyperlipidemia, or cardiovascular or endocrine diseases which could affect the autonomic nervous system such as pituitary insufficiency, thyroid disorders, and lack of any incident or surgery during the past months or during the study [36–39]. The method used for following up the participants is shown in Fig. 1.

Raw botanicals of EA (flowers of *Echium amoenum*) were purchased from Tehran drug/medicinal herbal market. They were then approved by Professor Gholamreza Amin at the Herbarium, Faculty of Pharmacy, Tehran University of Medical Sciences and kept under voucher number PMP-559. EA was crushed and extracted through the decoction method and was then freeze-dried; 150 mg of EA extract was granulated with Maize starch and were filled in 250 mg capsules. The capsules should be consumed three times a day, from the 21st day of one cycle to the 3rd day of the next cycle (10 days on aggregate) for two consecutive cycles. Placebo was prepared by filling only Maize starch in 250 mg capsules with the same shape as the EA capsules.

### Standardization of EA extracts

1. Total flavonoid content was determined using a routine reference standard method and the result was 17.3 Ru/g.
2. Total phenol content was determined using the Polin Ciocalteu’s method and the result was 3.48 mg GA/g.

The trial was conducted following the Declaration of Helsinki and following revisions [21] which were approved by the ethics committee of Tehran University of Medical Sciences; the study was registered in the Iranian Registry of Clinical Trials with the ID number of IRCT2015110822779N3, Registration date: 2015–11–27.

To check the normal distribution of the continuous variables, Kolmogorov-Smirnoff test was used; all the continuous data are shown as the mean ± standard deviation (SD). Independent samples t-test was used to compare the characteristics of the participants at the baseline between the placebo and the EA groups. Chi-square test was used for the categorical variables. It was approved that all the study variables had a normal distribution except for specific scores for symptoms of PMS and the total score.

Mann-Whitney and Friedman tests were used to compare the mean scores of the symptoms in the EA and the placebo groups according to the PSST before and after the first and the second cycles of the intervention. However, normality is not an essential assumption for Generalized Estimating Equation (GEE). Marginal modeling within GEE is considered as a powerful tool to analyze non-normally distributed data [40]. Therefore, GEE was used to compare the total scores of the intensity of PMS between the two study groups before and after the first and the second cycles of the intervention.

The level of statistical significance was set at any *p*-value below 0.05, based on 2-tailed tests premises. All the statistical analyses were performed using SPSS software version 20.0 for Windows (SPSS Inc., Chicago, USA).

## Results

The study flowchart is presented in Fig. 1. After randomization, 15 of all the eligible college students who participated in the present study, (5 in the EA group and 10 in the placebo group) were not willing to continue the study. In the placebo group, 10 participants did not finish the study; 4 due to using other drugs, 4 due to unwillingness to continue, and another 2 due to menstrual irregularity. In the EA group, 1 participant was excluded from the follow-up due to the misuse of EA capsules, and 4 due to being influenced by others’ opinions for using the treatment.

The baseline characteristics of the participants are reported in Table 1. No significant difference was observed between the two study groups regarding their baseline characteristics (*P* > 0.05).

**Table 1:** Comparison of base line characteristics between the two study groups

Characteristics	Placebo group *N* = 32	Echium amoenum group *N* = 37	*P*-value
^a^Age(years)	24.1 ± 2.7	24.5 ± 3.6	0.6
^a^BMI(kg/m^2^)	22.3 ± 2.3	22.4 ± 1.4	0.9
Marital status			
Never married;yes; N(%)	31(96.9)	36(97.3)	0.4
^a^Menarcheal age(year)	12.9 ± 1.4	13.3 ± 1.3	0.2
^a^ Duration of menstrual bleeding (day)	6.4 ± 1.1	6.5 ± 1.5	0.6
^a^Amount of bleeding (Number of vulva pads)	12.3 ± 4.2	14.2 ± 5.9	0.1
^a^Interval of menstrual cycle (days)	29.5 ± 2.8	28.9 ± 2.3	0.4
^a^Duration of PMS symptoms (days)	7.1 ± 3.5	5.7 ± 3.5	0.1
Dysmenorrhea;yes;n(%)	25(78.1)	34(91.9)	0.1
Familial history of PMS;yes;n(%)	22(68.8)	26(70.3)	0.2

^a^mean ± SD

*P*-value< 0.05 is statistically significant

Using PSST, the mean scores of the qualitative symptoms and their interference with daily activities of both groups, before and after the intervention, are reported in Table 2. The mean scores of different components of PSST in the study cycle were significantly lower compared to those before the treatment with EA. However, in the placebo group, the mean scores of components of PSST were not significantly lower than the scores before the intervention.

**Table 2:** Comparison of the mean rank of symptoms and intensity of complaints assessed by the Premenstrual Symptoms Screening Tool (PSST) in the Echium amoenum and placebo groups before and during the first and second cycle of intervention

PSST components	Placebo group *n* = 32 Mean rank				Echium amoenum group *n* = 37 Mean rank				**P-value
	Base line	1st cycle of intervention	2st cycle of intervention	*P-value	Base line	1st cycle of intervention	Second month	*P-value	
**Symptoms**									
1-Anger/irritability	42.4	40.6	39.5	0.07	46.8	36.7	24.6	**< 0.001**	**< 0.001**
2-Anxiety/tension	32.5	31.4	29.2	0.3	42.8	30.9	18.7	**< 0.001**	**< 0.001**
3-Tearful	33.2	30.0	29.8	0.3	42.6	31.5	18.6	**< 0.001**	**< 0.001**
4-Depressed mood	29.6	31.1	28.4	0.2	43.4	34.8	22.6	**< 0.001**	**< 0.001**
5-Decreased interest in work activities	29.5	25.9	25.0	0.1	39.8	28.8	20.2	**< 0.001**	**< 0.001**
6-Decreased interest in home activities	27.3	25.7	24.8	0.5	37.1	26.1	16.0	**< 0.001**	**< 0.001**
7-Decreased interest in social activities	26.7	23.1	21.9	0.2	40.3	27.8	19.0	**< 0.001**	**< 0.001**
8-Difficulty concentrating	36.4	34.3	32.8	0.1	34.7	30.4	22.3	**< 0.001**	**0.03**
9-Fatigue/lack of energy	37.7	35.4	32.9	0.1	43.2	31.8	20.2	**< 0.001**	**< 0.001**
10-Overeating/food cravings	20.4	18.8	18.6	0.6	27.3	24.4	16.9	**< 0.001**	**0.005**
11-Insomnia	15.3	12.5	14.7	0.7	24.1	18.4	13.7	**0.002**	**0.04**
12-Hypersomnia	31.1	29.1	26.9	0.1	35.4	21.4	17.9	**< 0.001**	**< 0.001**
13-Feeling overwhelmed	31.2	30.1	27.6	**0.2**	40.0	26.9	18.3	**< 0.001**	**< 0.001**
14-Physical symptoms	39.3	36.1	33.8	**0.1**	44.2	31.0	22.6	**< 0.001**	**< 0.001**
**Interference with**									**< 0.001**
15-work efficiency or productivity	33.2	29.5	26.8	0.09	40.8	30.2	19.8	**< 0.001**	**< 0.001**
16-relationships with coworkers	33.3	29.8	28.2	0.2	40.0	29.7	19.0	**< 0.001**	**< 0.001**
17-relationships with family	34.9	31.8	31.0	0.1	36.0	27.7	22.1	**< 0.001**	**0.02**
18-social life activities	28.8	25.1	26.1	0.2	37.2	29.6	25.4	**< 0.001**	**0.02**
19-home responsibilities	25.2	23.7	23.2	0.3	35.9	24.4	20.8	**< 0.001**	**0.003**
Total	31.0	29.4	28.3	0.09	35.3	24.2	16.1	< 0.001	< 0.001

^*^Within group *P*-value using Friedman test

^**^Between groups *P*-value using Mann-Withney test to compare the differences (baseline score-2nd follow up score) between two study groups

*P*-value < 0.05 considered statistically significant

As shown in Table 2, statistically significant differences were observed between all the components of PSST in both the treatment and the placebo groups. According to Table 2, the anxiety/tension and the tearful symptoms were the most associated symptoms, whereas the overeating/food cravings and the difficulty in concentrating symptoms were the least associated symptoms with the positive impact of EA consumption, respectively.

**Fig. 2:**
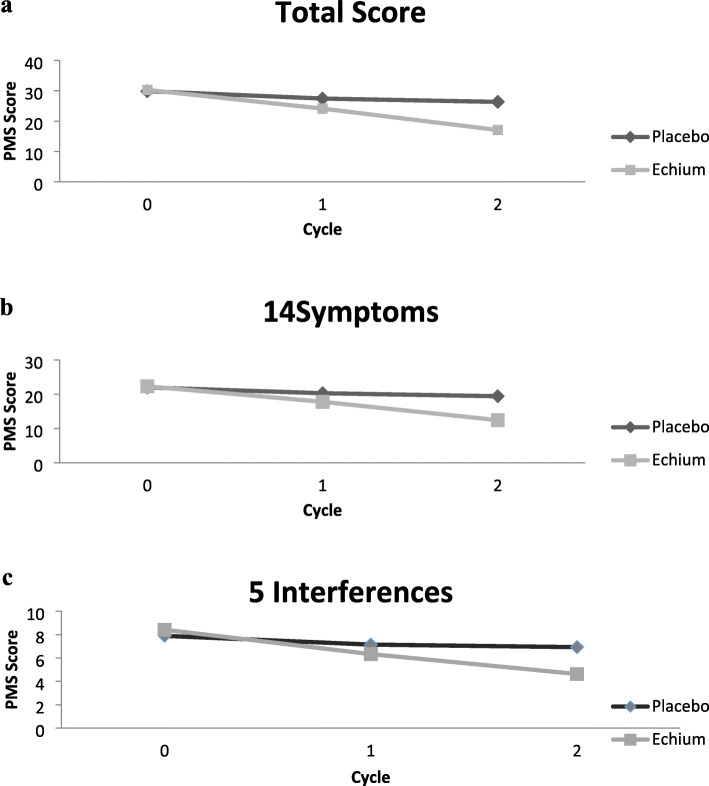
GEE estimated measures of the intensity of PMS according to the Premenstrual Symptoms Screening Tool (PSST), Total score (**a**), 14 symptoms (**b**), and 5 interferences with daily activities (**c**) in the Echium amoenum (EA) and the Placebo groups at 2 follow-ups regarding the interaction between time and the studied group and also adjusting for age and BMI. Patterns of mean changes differ between the EA group and the Placebo group

Based on GEE analysis during the follow-up, after adjustment for age and BMI variables, the overall mean score of the PMS was significantly higher in the placebo group than the EA group (Fig. 2).

Comparing the results of the first follow-up (cycle 1) demonstrated that the total scores of PMS, its 14 symptoms, and also its interference with various activities (according to PSST) in the EA group have decreased to 6.2, 4.4 and 1.3, respectively. Moreover, these values have decreased to 11.6, 8.5, and 2.8, respectively (Table 3) in the second follow-up (cycle 2) in the EA group.

**Table 3:** Parameter Estimates of PMS severity (total & subtotal) using GEE Model for study groups

Dependent Variables	Parameter	Beta	95% Confidence Interval	*P*-value
**Total score**	Echium amoenum	2.2	(−0.4,5.5)	0. 3
	Placebo	Ref.		
	Age(year)	0.09	(−3.4,0.5)	0.7
	BMI(kg/m^2^)	−0.5	(−1.2,0.2)	0.2
	2nd follow up visit	−3.8	(−5.8,-1.8)	**< 0.001**
	1st follow up visit	−4.0	(−5.9,-2.0)	**< 0.001**
	Baseline	Ref.		
	Echium amoenum^a^2nd follow up visit	−11.6	(− 14.7,-8.5)	**< 0.001**
	Echium amoenum^a^1st follow up visit	−6.2	(−9.8,-2.7)	**0.001**
	Placebo ^a^baseline	Ref.		
**Total score of symptoms**	Echium amoenum	2.1	(−0.3,4.6)	**0.2**
	Placebo	Ref.		
	Age(year)	−0.02	(− 0.3,0.3)	0.9
	BMI(kg/m^2^)	−0.3	(−0.8,0.2)	0.2
	2nd follow up visit	−2.8	(−4.5,-1.1)	**0.001**
	1st follow up visit	−2.9	(−4.5,-1.3)	**< 0.001**
	Baseline	Ref.		
	Echium amoenum^a^2nd follow up visit	−8.5	(−11.0,-6.1)	**< 0.001**
	Echium amoenum^a^1st follow up visit	−4.5	(−7.3,-1.8)	**0.001**
	Placebo ^a^baseline	Ref.		
**Total score of Interference with**	Echium amoenum	0.6	(−1.1,2.2)	0.5
	Placebo	Ref.		
	Age(year)	0.1	(−0.1,0.3)	0.3
	BMI(kg/m^2^)	−0.2	(−0.5,0.1)	0.2
	2nd follow up visit	−1.1	(−1.6,-0.5)	**< 0.001**
	1st follow up visit	−1.2	(−1.8,-0.5)	**< 0.001**
	Baseline	Ref.		
	Echium amoenum^a^2nd follow up visit	−2.8	(−4.1,-1.6)	**< 0.001**
	Echium amoenum^a^1st follow up visit	−1.3	(−2.7,0.07)	0.08
	Placebo ^a^baseline	Ref.		

*GEE* generalized estimating equation, *BMI* Body mass index

*P*-value< 0.05 is statistically significant

^a^Indicates interaction

## Discussion

The present study is the first, in the literature, to evaluate the safety and effectiveness of EA in comparison with placebo among women suffering from PMS. According to the findings, after the implementation of the intervention for two consecutive cycles, EA was more effective than a placebo in reducing the symptoms of PMS. Findings of the most recent studies have also shown that intervention with herbal medicine has reduced the symptoms of PMS [36–38, 41]; this can be explained by the fact that women who use therapeutic approaches have improved self-control over their lives, i.e., the psychological effects of the placebo can reduce the intensity of PMS [39].

PMS, which has a wide range of psychological, physical, and behavioral symptoms, is one of the most common health problems among women of reproductive ages and is highly prevalent worldwide. Women who are not willing to consume chemical drugs or who are dubious about consuming them might prefer herbal medicine for the treatment of PMS. And this can be considered as one of the main reasons why the use of herbal regimes is well-received in order to treat the symptoms of PMS in these groups [42, 43].

*EA,* known as “Gol-e-gavzaban” in Persian, is a plant that has not yet been reported to grow or be available in Europe or other parts of the world and is found exclusively in Iran [44]; it is one of the common traditional herbal medicines used as an effective treatment for skin disorders such as eczema, arthritis, diabetes, acute respiratory distress syndrome, alcoholism, obsessive-compulsive disorder, pain and swelling, and also to prevent heart diseases and stroke [23, 29, 45]. It is also used to treat bronchitis and colds and to help enhance sweating and increase breast milk flow and production. Traditionally, it has been used in hyperactive gastrointestinal and cardiovascular disorders [23]. Naturopathic practitioners use EA for the regulation of metabolism and the hormonal system [46]; they also consider it to be a good remedy for anxiety, PMS, and menopausal symptoms such as hot flash [25].

Flowers and the leaves are the main medicinal parts of the plant. The plant contains gamma-linolenic acid (GLA), alpha-linolenic acid (ALA), delta6-fatty acryl desaturase, delta8-sphingolipid desaturase, pyrrolizidine alkaloids, and mucilage, resin, potassium nitrate, calcium and mineral acids [27, 47, 48]. It seems that the functional mechanism of EA depends on a fatty acid called GLA. GLA might have anti-inflammatory effects, and so EA flower might have an antioxidant effect [25]. Studies have reported the useful effects of GLA on the reduction of the severity and duration of PMS symptoms [49]. In this regard, the results of a review study (2019) approved the efficacy of evening primrose oil, which is a rich source of GLA, in the reduction of severity of PMS symptoms after 4 to 6 months of consumption [50].

PMS has a complex series of behavioral, emotional and physical symptoms, and it is a documented fact that the majority of these symptoms are psychological; research has also shown elevated high-sensitivity C-reactive protein levels during PMS. In fact, among individuals with PMS, the transformation of inflammation from physiologic to pathologic state can increase the severity of PMS symptoms [51–53]. Furthermore, increased oxidative stress and decreased antioxidant capacity may occur in PMS [54]. As mentioned earlier, anti-inflammatory, analgesic, antioxidant, anti-anxiety, anxiolytic, anti-obsessive-compulsive, and antidepressant effects are among certain reported properties of EA [29]. The results of a study conducted by Sayyah et al. showed that EA is effective on certain psychiatric symptoms such as anxiety disorder, obsession-compulsion disorder, and depression without any severe side effects [29–31]; thereby, supporting the effectiveness of EA on reducing the severity of PMS symptoms.

Based on the findings of the present study, few side effects were reported by the participants; there were three cases of nausea in the placebo group, and none in the EA group and the findings were in line with those of other related studies [30, 31]. The researchers recommend that further studies should be conducted on active ingredients of EA to determine the effectiveness and safety of various doses and treatment sessions.

The present study had some limitations as well. First of all, EA was only administered for two cycles. Besides, the participants were college students and could not represent the general population of women with PMS. Self-report questionnaires were used to assess the intensity of PMS, which can affect the participant’s responses. Nevertheless, the main strength of this study was that it is the first research conducted on this topic and also the first to use validated PMS questionnaires.

## Conclusion

Considering the high prevalence of PMS among women of reproductive ages, effective treatments and safe strategies are highly recommended. EA has several therapeutic functions that can help reduce the severity of PMS and promote the health of women, along with its efficacy and safety in reducing the symptoms of PMS. Furthermore, conducting more studies is needed to assess the effects of EA on PMS to determine the active components, effectiveness, and safety of various doses with larger sample sizes using long-term interventions.

## Data Availability

The datasets generated and/or analyzed during the current study are not publicly available due to confidentiality considerations.

## Abbreviations

EA: Echium amoenum
PMS: Premenstrual syndrome
PSST: Premenstrual symptoms screening tool
GEE: Generalized estimating equation
GLA: Gamma-linolenic acid
ALA: Alpha linolenic acid
